# Hospital Expenditure—The Commissariat

**Published:** 1896-10-10

**Authors:** 


### The Institutional Workshop.

### HOSPITAL EXPENDITURE—THE COMMISSARIAT.

### XXIX.—MALT LIQUOR.

Nothing can illustrate more forcibly the growth of non-alcoholic principles, both in the treatment of disease and in the provisioning of a household, than the steady decline of this item of expenditure in hospitals. Some years ago an attempt was made to ascertain the value of the consumption of alcohol and milk in the leading hospitals during a period extending over twenty years, and it is thought that some of the information thus obtained may be usefully compared with the accounts for 1893, which have been lately under consideration. Wine and spirits, or "cordials," as they are termed in the Scotch hospitals, are now by the uniform system of accounts dismissed to the heading "surgery and dispensary." Malt liquor, including beer and porter, either bottled or in cask, is alone included under the heading of provisions.

The value of the figures for the preceding years is lessened by the want of data for estimating the number provisioned, whether patients or staff. It may, however, be taken as certain that the number of the hospital staff, including all those who are provisioned in the establishment has been at least doubled during the last thirty years, which have been marked by such striking changes in every department of hospital life. In several cases, too, the number of beds has been largely increased, and in no instance has there been any diminution in the number of patients boarded. The figures which give the value of the milk consumed in these years point to a very large increase in the number of persons boarded, the amount for 1885 being in every case more than double that for 1865, and in some cases as much as three or four times as much. It seems clear, then, that the use of malt liquor, both as a form of tonic for convalescent patients and as an article for food for men and women actively employed, is steadily declining in institutions.

Expenditure on Malt Liquor.

| | 1865. | | | 1875. | | | 1885. | | |
|---|---|---|---|---|---|---|---|---|---|
| | Staff. | Ptnts. | Tl. | Staff. | Ptnts. | Tl. | Staff. | Ptnts. | Tl. |
| | £ | £ | £ | £ | £ | £ | £ | £ | £ |
| Middlesex | — | — | 353 | — | — | 532 | 221 | 95 | 316 |
| St. Mary's | — | — | 428 | — | — | 185 | — | — | 157 |
| St. George's | 269 | 367 | 636 | 330 | 230 | 560 | 300 | 140 | 440 |
| (The number of the nursing staff increased from 54 in 1865 to 100 in 1885.) | | | | | | | | | |
| University | — | — | 195 | — | — | 250 | — | — | 213 |
| (Hospital enlarged in 1879 by 46 beds.) | | | | | | | | | |
| Seamen's | — | — | 85 | — | — | 195 | — | ... | 166 |
| King's College | — | — | 362 | — | — | 160 | 63 | 19 | 82 |
| Charing Cross | 17 | 112 | 129 | 72 | 120 | 192 | 35 | 50 | 84 |
| London | — | — | 904 | — | — | 905 | 389 | 349 | 738 |
| Royal Free | 26 | 189 | 215 | 34 | 138 | 172 | 55 | 70 | 115 |
| (Beds and staff increased 30 per cent. since 1875 by addition of new wing.) | | | | | | | | | |
| Metropolitan | — | — | 64 | — | — | 124 | — | — | 16 |
| (Hospital closed in 1885 for nine months.) | | | | | | | | | |

Side by side with this table should be studied the expenditure on this item for 1893, in which year the number of inmates having been accurately ascertained, it has been possible to calculate the average yearly amount expended in each hospital per head. It may be noted that as 1s. per gallon, or 36s. per barrel, is the common price paid in London hospitals for the household beer, each £1 represents a total of 320 half pints or glasses of beer. Reckoning, therefore, that at least half the inmates at any given time are not beer drinkers, these figures show that about half a glass a day falls in most instances to the remainder, while in no case is the daily allowance much over half a pint. It is not, of course, possible to reach any conclusion as to the actual amount of malt liquor consumed by each person, as may be judged by a glance at the different proportion spent in different hospitals on beer for patients and staff. In no particular are customs more diverse. Yet it is only by striking this sort of rough average per head that it becomes possible to prove the very small extent to which malt liquor is consumed at the present time in these institutions.

The figures at Guy's Hospital shows a yearly average for patients of 1s. per head, and for the household of 13s. per head. At the Birmingham General, the quantity consumed averages for patients three pints each in the year, and for the household 264 pints each. At the Great Northern Central the yearly average is 1s. 7d. each for patients, and 2s. for each member of the household.

EXPENDITURE ON MALT LIQUOR, 1893.

| | General average per head. | Total Expenditure. | Difference since 1865. | Difference since 1885. |
|---|---|---|---|---|
| | | £ | £ | £ |
| Middlesex | 15s. | 276 | 77 decrease | 40 decrease |
| St. Mary's | 12s. | 238 | 190 ,, | 81 increase |
| St. George's | £1 | 511 | 125 ,, | 71 ,, |
| University | 10s. | 124 | 71 ,, | 89 decrease |
| Seamen's | 17s. | 251 | 163 increase | 85 increase |
| King's College | — | 129 | 233 decrease | 47 ,, |
| Charing Cross | 15s. | 182 | 52 increase | 97 ,, |
| London | 17s. | 652 | 252 decrease | 86 decrease |
| Royal Free | 8s. | 72 | 143 ,, | 53 ,, |
| Metropolitan | 1s. 4d. | 7 | 57 ,, | 9 ,, |

It will be seen that, with two exceptions, all these hospitals have decreased their expenditure on malt liquor since 1865 to an extent almost incredible when the enormous increase in every case of the number boarded is taken into consideration. Lest it should be thought that other alcoholic drinks may have taken the place of malt liquor, it must be noted that in almost every instance the consumption of wine and spirits has diminished to a corresponding extent. At the London, for instance, the wine and spirit bill sank from £1,462 in 1865 to £525 in 1885, and at University from £334 to £183. And many similar examples might be quoted.

Between 1885 and 1893 the reduction in the amount of alcohol consumed is not so marked, since institution housekeeping has not undergone during this period the radical changes which were a feature of the preceding twenty years. Yet here, again, the tendency is to a steady decrease, the small rise in some hospitals being amply accounted for by an increase in the number of beds and staff.

The same decrease appears to have been going on all over the country. At the Bristol General the amount expended on malt liquor fell from £153 in 1865 to £97 in 1885; at the Manchester Royal Infirmary the fall was from £238 to £148; the fall in the cost of wine and spirits during the same period of twenty years being from £562 to £52. At the Lincoln County Hospital the cost of malt liquor was £128 in 1865, and £49 in 1885. These instances might be multiplied indefinitely.

One important point calls for attention in this connection. In one or two cases mention is made in the report of the fact that an allowance in lieu of beer is made to members of the staff, which is included under the item of wages and salaries. In the case of the Devon and Exeter Hospital, the cost of malt liquor is only £158 in 1885 as compared with £367 in 1865 and 1875, and the cause for this reduction is expressly stated to be that the staff now receive a money consideration in lieu of beer. It need scarcely be said that where a sum of money is given for the purchase of any article of food, the sums thus expended should be included among provisions instead of salaries. At Halifax Infirmary the very modest sum total of £42 expended on malt liquor is assigned to "beer and beer allowance," and where these allowances are not thus included the results cannot be said to be quite fairly estimated. At the same time, it is not always possible to define very crisply the various distinctions in custom which arise in these strangely varied households, and in cases where all salaries have been permanently raised on the understanding that malt liquor is not provided, the beer element cannot always be disentangled. Extra payments made to individuals on the strength of their not receiving malt liquor provided for other members of the household should invariably be reckoned, however, as though the money were actually spent on beer.

In provincial institutions, as may be seen below, the yearly average for malt liquor is in no case more than 12s. or 13s. per head, and the minimum approaches a vanishing quantity.

YEARLY AVERAGE EXPENDITURE PER HEAD ON MALT LIQUOR.

| | s. | d. | | s. | d. |
|---|---|---|---|---|---|
| Norfolk and Norwich | 14 | 0 | Leeds... ... ... | 4 | 0 |
| Sheffield General ... | 13 | 0 | Newcastle-on-Tyne... | 4 | 0 |
| Oxford Radcliffe ... | 12 | 0 | Hull County ... | 3 | 0 |
| Sussex County ... | 12 | 0 | York County ... | 1 | 0 |
| Birmingham General | 8 | 0 | Glasgow Royal ... | 5 | 0 |
| Birmingham Queen's | 7 | 0 | *Edinburgh... ... | 1 | 6 |
| Bristol General ... | 7 | 0 | Aberdeen ... ... | 1 | 0 |
| Halifax ... ... | 6 | 0 | Glasgow Western ... | 0 | 6 |
| Cambridge Addenbrooke ... ... | 4 | 0 | Belfast Royal ... | 2 | 0 |

* Including aerated waters.

There is more variation in the price paid for beer than in London. The higher prices are 40s., 42s., 45s., 48s., and even 60s. per barrel, while the price drops in several places to 27s., 26s., 24s., and 22s. 6d.

